# A patent review of cyclin-dependent kinase 5 (CDK5) inhibitors (1999–2025)

**DOI:** 10.3389/fbioe.2026.1843047

**Published:** 2026-06-02

**Authors:** Varvara M. Derzhavina, Igor A. Khalymbadzha, Arseniy E. Yuzhalin

**Affiliations:** Research Center for Translational Medicine, Sirius University of Science and Technology, Sochi, Russia

**Keywords:** cyclin-dependent kinase 5, dinaciclib, inhibitor, milciclib, neurodegeneration, patent, purvalanol A, roscovitine

## Abstract

Cyclin-dependent kinase 5 (CDK5) is a critical regulator of neuronal development and function, whose hyperactivation exacerbates neurodegenerative disorders and certain cancers. While CDK5 is a compelling therapeutic target, achieving selective inhibition is challenging due to high structural homology with cell-cycle CDKs and poor blood-brain barrier penetrance. The article provides the first comprehensive analysis of patented CDK5 inhibitors disclosed between 1999 and 2025. We examine the chemical diversity, selectivity profiles, and therapeutic claims of major chemotypes, including purine analogs (e.g., roscovitine), pyrazole derivatives (e.g., dinaciclib, milciclib), indolobenzazepinones, indirubin derivatives, and emerging modalities such as peptides. Furthermore, we discuss major obstacles such as overcoming off-target toxicity against other CDKs, ensuring sufficient CNS exposure, and identifying reliable biomarkers. Lastly, we speculate on how future success will depend on new strategies such as p25-specific modulation, targeted protein degradation, and advanced delivery systems to translate CDK5′ therapeutic potential into the clinic.

## Highlights

- CDK5 is a crucial kinase in neuronal physiology, but its hyperactivation is a pathogenic driver in major neurodegenerative diseases (Alzheimer’s, Parkinson’s) and certain CNS cancers, establishing it as a high-value therapeutic target.
- Developing selective CDK5 inhibitors is exceptionally challenging due to high structural homology in the ATP-binding pocket with cell-cycle CDKs (CDK1/2), leading to off-target toxicity, and the restrictive blood-brain barrier limiting CNS drug delivery.
- The patent landscape (1999–2025) reveals diverse chemotypes, from early pan-CDK inhibitors (purine analogs like roscovitine, pyrazoles like dinaciclib) to newer, more selective strategies, including CDK5-directed inhibitory peptides designed to counteract p25-mediated neurodegenerative signaling.
- While numerous inhibitors show preclinical efficacy, none have achieved clinical approval, primarily due to insufficient selectivity, suboptimal pharmacokinetics, and a lack of validated biomarkers for target engagement.
- The future of CDK5 therapeutics will depend on innovative approaches such as allosteric/p25-selective inhibition, proteolysis-targeting chimeras (PROTACs), and advanced CNS delivery systems to achieve clinical success in treating neurodegeneration and cancer.

## 1 Introduction

Cyclin-dependent kinases (CDKs) constitute a highly conserved family of serine/threonine kinases that play pivotal roles in regulating cell cycle progression, transcription, metabolism, and neuronal function. The human genome encodes over 20 CDKs, which are traditionally classified by their primary physiological roles: cell-cycle CDKs (e.g., CDK1, CDK2, CDK4/6), transcriptional CDKs (e.g., CDK7, CDK8, CDK9), and atypical CDKs such as CDK5. CDK5 is not activated by cyclins but instead by binding to neuron-specific activators p35 and p39 (and their cleaved forms p25 and p29) (Shupp et al., 2017; Sharma and Sicinski, 2020; Batra et al., 2023; Pluta et al., 2024).

Given their central role in proliferation and disease, CDKs have long been pursued as therapeutic targets, particularly in oncology (Ghafouri-Fard et al., 2022). Several pan-CDK inhibitors (e.g., flavopiridol, dinaciclib, seliciclib) have advanced to clinical trials, but their development has been hampered by dose-limiting toxicities and off-target effects arising from poor isoform selectivity (Benson et al., 2007; National Cancer Institute NCI, 2013; Merck Sharp and Dohme LLC, 2017; Bansal et al., 2023). While highly selective inhibitors exist for certain CDKs (e.g., CDK4/6 inhibitors like palbociclib), CDK5 remains a challenging target due to structural similarities within the kinase family and a lack of truly specific small-molecule inhibitors (Kaveh et al., 2024; PubChem, 2006a). This pharmacological gap underscores the need for compounds that can selectively modulate CDK5 activity without disrupting other essential CDK pathways.

CDK5 activity is predominantly restricted to the nervous system due to the neuron-specific expression of CDK5’s activators (Do and Lee, 2021; Pao and Tsai, 2021; Batra et al., 2023). Early *in vivo* studies revealed that mouse pups with whole-body CDK5 or p35/p39 knockout display 100% perinatal lethality with severe defects in neuronal layering due to impaired migration (Lopes and Agostinho, 2011; Shupp et al., 2017; Sharma and Sicinski, 2020). These works clearly demonstrated that CDK5 controls neural processes such as neuronal migration during brain development, neurite outgrowth, dendritic arborization, axonal elongation, *etc.* (Kesavapany et al., 2004; Sharma and Sicinski, 2020; Łukasik et al., 2021) CDK5 also regulates cytoskeletal dynamics by phosphorylating a wide array of neuronal proteins comprising the cytoskeleton, which regulates neuronal shape and synaptic function (Shah and Rossie, 2018; Jahan et al., 2023; Fu et al., 2025).

In neurological diseases, aberrant activation of CDK5 is not uncommon and can be associated with disease progression. In Alzheimer’s disease (AD), CDK5 hyperactivation leads to overphosphorylation of pathologically relevant substrates such as tau and amyloid precursor protein, contributing to the emergence of hallmark AD features and ultimately leading to clinical manifestations of AD (Hisanaga et al., 2022; Fu et al., 2025) CDK5 hyperactivity also promotes neuronal cell cycle re-entry, a step linked to neurodegeneration in AD (Liu et al., 2016; Allnutt et al., 2020). In Parkinson’s disease (PD) CDK5/p25 hyperactivation, triggered by dopaminergic toxins like MPTP or α-synuclein aggregates, phosphorylates a plethora of substrates such as tau (promotes aggregation), DARPP-32 (alters dopamine signaling), and peroxiredoxin-2 (exacerbates oxidative stress), all leading to nigrostriatal degeneration (Bogush et al., 2007; Binukumar et al., 2015; Nishi and Shuto, 2017; Ao et al., 2022; Martinez-Banaclocha, 2023; Alrouji et al., 2024).

Beyond neurodegeneration, CDK5 is overexpressed and functionally implicated in certain CNS cancers. In glioblastoma (GBM) patients, higher CDK5 expression correlates with tumor grade and progression, potentially through influencing the STAT3 pathway *via* substrates like TRIM59 (Binukumar et al., 2015; Sang et al., 2019; Zhou et al., 2021). CDK5 hyperactivation drives medulloblastoma metastases by promoting immune evasion, cytoskeletal remodeling, and invasive phenotypes, particularly in sonic hedgehog and group 3 subtypes prevalent in children. Mechanistically, CDK5/p35 phosphorylates PD-L1 at multiple sites, which leads to stabilized surface expression of this immune checkpoint, thus enabling immune escape (Yuzhalin et al., 2024).

Targeting CDK5 is challenging due to its high structural similarity to cell-cycle CDKs, particularly CDK1 and CDK2, with which it shares 58%–62% sequence homology. All three kinases exhibit conserved catalytic domains characterized by similar ATP-binding pockets and substrate-binding sites, which makes the design of selective inhibitors difficult (McGrath et al., 2017; Wood and Endicott, 2018; Teng et al., 2020).

A major obstacle in targeting CDK5 for CNS diseases stems from the blood-brain barrier (BBB), which restricts the penetration of most small-molecule inhibitors. Existing CDK5 inhibitors (e.g., roscovitine, PHA-767491) often exhibit poor CNS bioavailability due to high molecular weight, polarity, or efflux by transporters such as P-glycoprotein, which hampers clinical translation (Vita et al., 2005; Rojas-Prats et al., 2021; 2021). Whereas certain brain-penetrant compounds, such as TAT-conjugated peptides, demonstrate BBB crossing and neuroprotection in animal models of neurodegeneration, no CDK5-targeted agent has advanced to approved clinical use (Mushtaq et al., 2016; Umfress et al., 2022; Pao et al., 2023; Tsai and Seo, 2025; Cdk5 inhibitors: Combating Cognitive Disorders, n.d.). This situation underscores the need for developing new effective compounds.

CDK5 inhibitor selectivity is complicated by conserved ATP-binding pockets shared with mitotic CDKs (CDK1/CDK2), leading to off-target cell cycle arrest and cytotoxicity in dividing cells. Pan-CDK inhibitors may effectively suppress the CDK5/p25 hyperactivity driving neurodegeneration, but they also induce apoptosis in healthy dividing cells, which raises toxicity concerns. In addition, since physiological CDK5 maintains neuronal quiescence through Rb and p21 phosphorylation, selective inhibition requires allosteric or p25-specific modulators to preserve these functions while blocking hyperactivation (Zhang et al., 2012; Allnutt et al., 2020; Tang et al., 2020; Requejo-Aguilar, 2025).

While CDK5 has been extensively studied, its intellectual property (IP) landscape has not been systematically evaluated in light of recent therapeutic paradigm shifts. Early patents predominantly claimed ATP-competitive scaffolds with broad CDK cross-reactivity and limited CNS penetration. In contrast, contemporary filings increasingly emphasize context-selective inhibition, BBB-optimized small molecules, peptide-based complex disruptors, and targeted protein degradation. However, a critical gap remains: many patents assert broad CDK5 inhibition without corresponding *in vivo* validation, claim overlap creates freedom-to-operate (FTO) uncertainty, and the alignment between IP strategy and clinically relevant biological targets (e.g., CDK5/p25 vs. CDK5/p35) remains poorly mapped (Pant et al., 2013; Teng et al., 2020; Gray et al., 2022; Umfress et al., 2022; Pao et al., 2023). This review addresses this gap by cataloging and critically analyzing recent patent activity, differentiating truly novel claims from incremental variations, and highlighting underprotected mechanistic niches that could accelerate the development of next-generation CDK5 therapeutics.

## 2 Analysis of patented CDK5 inhibitors (1999–2025)

### 2.1 Purine derivatives

**FIGURE 1:**
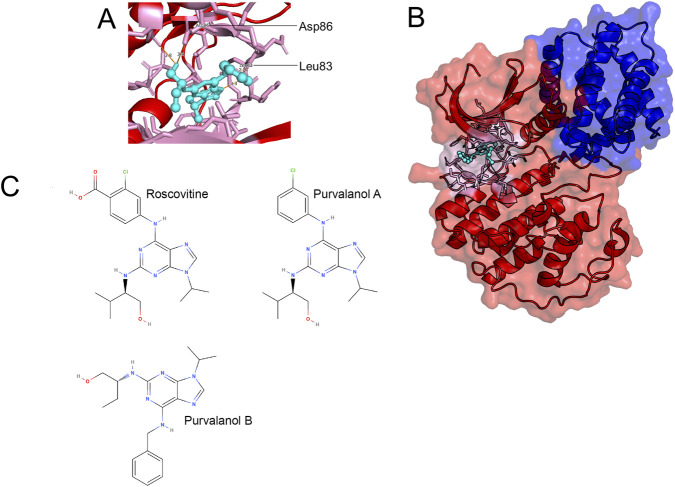
Structural basis of CDK5/p25 inhibition by purine derivatives. **(A)** Close-up view of the ATP-binding pocket of the CDK5/p25 complex (chain A: red; chain D: blue) with roscovitine (cyan) bound. Key catalytic residues Asp86 and Leu83 are shown in pink sticks, forming hydrogen bonds (orange lines, distances in Å) with the inhibitor. The surrounding protein surface is rendered semi-transparently in pink to highlight the binding cleft. **(B)** Global view of the CDK5/p25–roscovitine complex. Chain A (CDK5) is depicted as red ribbons, chain D (p25) as blue ribbons, and the molecular surface is shown in pink to delineate the active site region. Roscovitine (cyan) occupies the canonical ATP-binding site between the N- and C-terminal lobes, consistent with its mechanism as a competitive kinase inhibitor. **(C)** Chemical structures of representative purine analogs: roscovitine, Purvalanol A, Purvalanol B. 3D model created using SwissDock.

Roscovitine (Figures 1A–C), also known as 2-(R)-(1-Ethyl-2-hydroxyethylamino)-6-benzylamino-9-isopropylpurine or CYC202/Seliciclib, represents a foundational trisubstituted purine derivative among early-generation CDK inhibitors, initially developed through structure-activity optimization of the adenine-based compound olomoucine to enhance potency and selectivity toward cell cycle regulators (Griffin et al., 1999; Oumata et al., 2008). Characterized by a (R)-(1-hydroxybut-2-yl)amino) substituent at the C-2 position, a benzylamino group at C-6, and an isopropyl moiety at the N-9 position of the purine core, roscovitine binds competitively in the ATP-binding pocket of CDKs, forming key hydrogen bonds with hinge region residues such as Leu83 (CDK2, PDB: 2A4L) and exhibiting micromolar potency against CDK1s/cyclin B (IC_50_ = 100–650 nM), CDK2/cyclin A/E (IC_50_ = 0.7 μM), and CDK5/p25 while displaying reduced activity against CDK4/6 (IC_50_ > 10 μM) and broader kinome selectivity relative to non-CDK kinases (Oumata et al., 2008). This pharmacological profile induces G1/S and G2/M cell cycle arrest, apoptosis *via* Mcl-1 downregulation, and inhibition of RNA polymerase II CTD phosphorylation through off-target effects on transcriptional CDKs (e.g., CDK7/9), contributing to antiproliferative efficacy in preclinical models of breast, lung, and hematological cancers with GI_50_ values typically in the 5–20 μM range across diverse cell lines (Bettayeb et al., 2010). Despite promising antitumor activity in xenograft models and advancement to phase II/III clinical trials [e.g., NCT03134638 for NSCLC and breast cancer (Clinical Trial: NCT03134638 - My Cancer Genome, n.d.)], roscovitine’s polypharmacology, modest selectivity, and suboptimal pharmacokinetics limited its clinical success, inspiring subsequent pyrazolo [1,5-a]pyrimidine analogs like BS-181 and samuraciclib for improved CDK7 specificity. Early patents, such as WO1999002162A1, exemplify IP claims around 2,6,9-trisubstituted purines for CDK inhibition in proliferative disorders, underscoring roscovitine’s role as a privileged scaffold in the evolution of selective CDK modulators (Griffin et al., 1999; Cicenas et al., 2015; Kovalová et al., 2023).

Purvalanols A and B (Figure 1C) are second-generation purine CDK inhibitors derived from the original roscovitine scaffold by removal of the methylene linker from the benzylamino moiety and systematic SAR optimization, yielding tighter engagement of the conserved ATP-binding cleft and improved potency and kinome selectivity toward CDK1/2 (and CDK5 at higher concentrations) (Albrecht et al., 2002). Purvalanol A inhibits Cdc2/CDK1–cyclin B with low-nanomolar potency and displays a well-characterized activity profile against CDK1, CDK2, and CDK5, translating into robust G1/S and G2/M blockade, suppression of Rb hyperphosphorylation, caspase activation, and apoptosis across multiple tumor models, including lung, colon, prostate, leukemia, and cisplatin-resistant ovarian cancer cells (Sakurikar and Eastman, 2016; Chen et al., 2017; Zhang et al., 2023; P-2481-10MG - Purvalanol A, 10 MG, n.d.). This multi-target profile also extends to inhibition of c-Src signaling, where purvalanol A suppresses Src activity as effectively as PP2 while inducing a strong G2/M arrest and reversing Src-driven transformation (Hikita et al., 2010). *In vivo*, purvalanol A demonstrates antitumor efficacy in xenograft settings (for example, enhancement of taxol-induced apoptosis and clonogenic loss in irradiated gastric cancer models), but its poor aqueous solubility and suboptimal pharmacokinetics have prevented progression beyond preclinical development (Obakan et al., 2014; Zhang et al., 2023; Yang et al., 2026).

### 2.2 Pyrazoles derivatives

**FIGURE 2:**
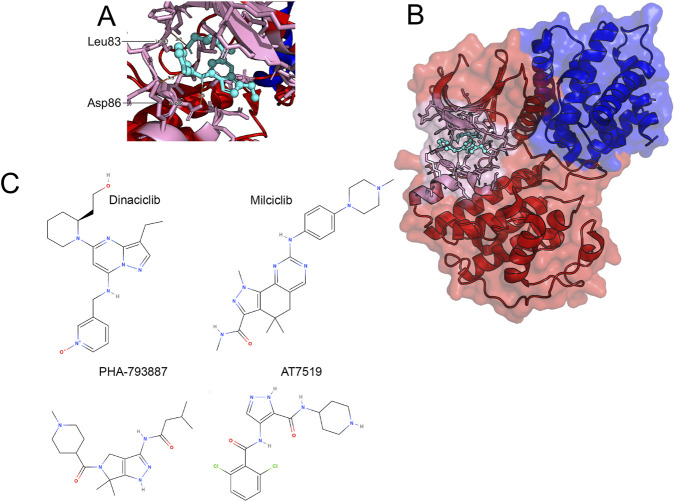
Structural basis of CDK5/p25 inhibition by pyrazole-based kinase inhibitors. **(A)** Close-up view of the ATP-binding pocket of the CDK5/p25 complex (chain A: red; chain D: blue) with Dinaciclib (cyan) bound. Key residues Leu83 and Asp86 (pink sticks) form hydrogen bonds (orange lines, distances in Å) with the inhibitor’s heterocyclic core and substituents. The surrounding protein surface is rendered semi-transparently in pink to highlight the binding cleft. **(B)** Global view of the CDK5/p25–Dinaciclib complex. Chain A (CDK5) is depicted as red ribbons, chain D (p25) as blue ribbons, and the molecular surface is shown in pink to delineate the active site region. Dinaciclib (cyan) occupies the canonical ATP-binding site between the N- and C-terminal lobes, consistent with its mechanism as a competitive ATP-mimetic inhibitor. **(C)** Chemical structures of representative pyrazole derivatives: Dinaciclib, Milciclib, PHA-793887, AT7519. 3D model created using SwissDock.

Dinaciclib (SCH727965) (Figures 2A–C) is a synthetic small-molecule inhibitor belonging to the pyrazolo[1,5-a]pyrimidine class of heterocycles (Guzi et al., 2006; Dinaciclib (SCH 727965) | CDK Inhibitor | CAS 779353-01-4 | Selleck Chemicals, n.d.). Pyrazolo [1,5-a]pyrimidine core can be derived from purine by bioisosteric substitution. It acts as a potent ATP-competitive inhibitor of multiple cyclin-dependent kinases, including CDK1, CDK2, CDK5, CDK9, and CDK12, with low nanomolar inhibitory concentrations (Dinaciclib, cyclin-dependent kinase (CDK) inhibitor (CAS 779353-01-4)|Abcam, n.d.; Dinaciclib (SCH 727965)|CDK Inhibitor|CAS 779353-01-4|Selleck Chemicals, n.d.). Structurally, dinaciclib binds within the ATP-binding pocket of CDK2 (PDB: 4KD1) with a key hydrogen bond between a pyrazole nitrogen and the hinge region backbone amide of Leu83. Additional polar contacts with residues in the glycine-rich loop (e.g., Lys33) and αC-β4 region (e.g., Lys89) may contribute to binding affinity and selectivity, though these interactions are likely water-mediated or conformation-dependent (Martin et al., 2013; Chohan et al., 2018; Roskoski, 2019; Hope et al., 2025). The compound demonstrates potent inhibition of CDK-mediated phosphorylation events leading to cell cycle arrest at G1/S and G2/M phases, downregulation of transcriptional elongation through CDK9 blockade, and induction of apoptosis in various cancer cell lines (Johnson et al., 2012). Dinaciclib has shown promising preclinical efficacy in xenograft models of leukemia, multiple myeloma, and solid tumors, and has advanced into multiple clinical trials for hematological and solid malignancies. Its favorable pharmacokinetic properties and manageable safety profile support ongoing investigation as a therapeutic option targeting cell cycle dysregulation and transcriptional dependencies in cancer (Johnson et al., 2012; Kumar et al., 2015; Gregory et al., 2022; Beltrán-Visiedo et al., 2023).

PHA-793887 (Figure 2C), systematically named N-(6,6-dimethyl-5-((1-methylpiperidin-4-yl)carbonyl)-1,4,5,6-tetrahydropyrrolo [3,4-c]pyrazol-3-yl)-3-methylbutanamide (CAS 718630-59-2), embodies an optimized 6,6-dimethyl-1,4,5,6-tetrahydropyrrolo [3,4-c]pyrazole scaffold among third-generation pan-CDK inhibitors, arising from medicinal chemistry refinement at Nerviano Medical Sciences to balance potency, physicochemical properties, and intravenous tolerability for oncology applications (Brasca et al., 2008; 2010; PHA-793887|718630-59-2|CDK|MOLNOVA, n.d.; PHA-793887|CDK inhibitor|CAS 718630-59-2|Selleck, n.d.; PHA-793887|CDK2/5/7 inhibitor, n.d.). The bicyclic core, gem-dimethyl substituted at C-6 to rigidify the pyrrolidine ring, bears a 3-methylbutanamido group at C-3 for hydrogen bonding to the kinase hinge (e.g., Leu83 in CDK2, PDB: 2WPA), a (1-methylpiperidin-4-yl)carbonyl at C-5 exploiting the ribose pocket, and a fused pyrazole enabling additional hydrophobic interactions, delivering subnanomolar to low nanomolar inhibition across CDK2/cyclin A (IC_50_ = 5 nM), CDK1/cyclin B (IC_50_ = 9 nM), CDK5/p25 (IC_50_ = 10 nM), and CDK7 (IC_50_ = 20 nM), with moderate selectivity over CDK4/6 (IC_50_ > 100 nM) and broader kinome profiling revealing off-targets like GSK3β at higher concentrations (PHA-793887|718630-59-2|CDK|MOLNOVA, n.d.; PHA-793887|CDK Inhibitor|MedChemExpress, n.d.). This multi-CDK blockade elicits potent G1/S arrest, pRb hypophosphorylation, caspase-3/7 activation, and apoptosis in solid tumor lines (e.g., A2780 GI_50_ = 50 nM, HCT-116 GI_50_ = 80 nM, BXPC-3 GI_50_ = 120 nM), translating to robust xenograft regressions (>80% TGI) in ovarian, colon, and pancreatic models upon daily IV dosing (20–40 mg/kg) with favorable tolerability (no significant body weight loss) and linear pharmacokinetics (T_1/2_–2 h in rodents) (Alzani et al., 2010; PHA-793887|718630-59-2|CDK|MOLNOVA, n.d.). Though advanced to phase I/II trials (e.g., NCT00970834 for advanced solid tumors) before discontinuation due to overlapping profiles with emerging isoform-selectives, PHA-793887 exemplifies early efforts in constrained pyrazole chemotypes influencing later designs like pyrazolo [1,5-a]pyrimidines, with IP protected in Nerviano filings (e.g., WO2006/080376 equivalents) claiming pyrrolopyrazole CDKs for proliferative diseases (Alzani et al., 2010; Brasca et al., 2010; PHA-793887|CDK inhibitor|CAS 718630-59-2|Selleck, n.d.).

AT7519 (Figure 2C), chemically 4-(2,6-dichlorobenzamido)-N-(piperidin-4-yl)-1H-pyrazole-3-carboxamide (CAS 902135–91-5), constitutes a second-generation aminopyrazole-based multi-CDK inhibitor discovered by Astex Therapeutics *via* fragment-based drug design coupled with X-ray crystallography, exemplifying ATP-competitive agents optimized for broader kinome coverage in proliferative disorders (Berdini et al., 2008; PubChem, 2005a; AT7519|CDK inhibitor, n.d.). The core pyrazole-3-carboxamide scaffold, forms canonical hinge hydrogen bonds (e.g., with Leu83 in CDK2, PDB: 2VU3) while the chlorophenyl and piperidine moieties occupy hydrophobic regions adjacent to the gatekeeper, affording low nanomolar potency against CDK1/cyclin B (IC_50_ = 220 nM), CDK2/cyclin A/E (IC_50_ = 44–194 nM), CDK4/6 (IC_50_ = 170–340 nM), CDK5 (IC_50_ = 100–300 nM), CDK7 (IC_50_ = 500 nM), and CDK9 (IC_50_ = 45 nM), with >10-fold selectivity over non-CDK kinases in panels up to 100 members and reduced activity against CDK3 (Chen et al., 2014; AT7519|CDK inhibitor, n.d.; AT7519 Hydrochloride|CDK Inhibitor|MedChemExpress, n.d.). This polypharmacology suppresses RNA polymerase II CTD phosphorylation (*via* CDK7/9), induces rapid G1/S and G2/M arrest, Rb hypophosphorylation, Mcl-1 downregulation, GSK-3β activation, and apoptosis in diverse tumor cells (GI_50_ = 0.1–1 μM in colon, breast, myeloma, and GBM lines), yielding tumor regressions (>70% TGI) in HCT116, HT29, and myeloma xenografts upon IV/oral dosing (5–15 mg/kg BID) with acceptable pharmacokinetics (T_1/2_ ∼3–5 h, moderate clearance). Advanced to phase I/II trials (e.g., NCT00312262, NCT00920213 for solid tumors and hematological malignancies) before discontinuation owing to myelosuppression and suboptimal efficacy relative to isoform-selectives, AT7519 HCl’s hydrochloride salt enhances solubility for IV administration (Squires et al., 2009; Santo et al., 2010; Chen et al., 2014; Xi et al., 2019; Zhao et al., 2023).

Milciclib (PHA-848125) (Figure 2C) is a potent orally bioavailable small molecule inhibitor characterized by a 1H-pyrazolo [4,3-h]quinazoline scaffold, featuring dihydro-N,1,4,4-tetramethyl substitution and decorated with a 4-(4-methylpiperazin-1-yl)phenylamino moiety that provides kinase-binding specificity and favorable pharmacokinetics (Traquandi et al., 2009; Milciclib (PHA-848125)|CDK inhibitor|CAS 802539-81-7|Buy Milciclib (PHA848125) from Supplier InvivoChem, n.d.; Milciclib (PHA-848125)|CDK/TRK Inhibitor|MedChemExpress, n.d.). As a CDK inhibitor, milciclib potently inhibits CDK2/cyclin A with reported IC_50_ values around 45 nM and also affects multiple CDK family members including CDK1, CDK4, CDK5, as well as tropomyosin receptor kinase A, thereby modulating cell cycle progression, DNA replication, and cell signaling pathways. Specifically regarding CDK5, milciclib demonstrates moderate inhibitory activity, helping suppress CDK5-mediated phosphorylation pathways that are implicated in diverse cancers and neurodegeneration contexts, though detailed IC_50_ values for CDK5 inhibition typically exceed those for CDK2 (Milciclib (PHA-848125)|CDK inhibitor|CAS 802539-81-7|Buy Milciclib (PHA848125) from Supplier InvivoChem, n.d.; PHA-848125|CAS NO.:802539-81-7|GlpBio, n.d.). Functionally, milciclib induces cell cycle arrest at G1/S and G2/M phases, inhibits Rb phosphorylation, and promotes apoptosis across multiple cancer cell lines, including breast and thyroid cancers, with preclinical *in vivo* efficacy (Milciclib|CDK inhibitor|CAS 802539-81-7|Selleck, n.d.; Milciclib (PHA-848125)|CDK inhibitor|CAS 802539-81-7|Buy Milciclib (PHA848125) from Supplier InvivoChem, n.d.). Clinical investigations have explored milciclib’s anticancer potential in phase II trials, leveraging its multitarget kinase inhibition profile; however, its precise CDK5-related therapeutic role remains under evaluation (Tiziana Life Sciences plc, 2019; Wells et al., 2020; Milciclib (PHA-848125)|CDK inhibitor|CAS 802539-81-7|Buy Milciclib (PHA848125) from Supplier InvivoChem, n.d.).

### 2.3 Indolobenzazepinones

**FIGURE 3:**
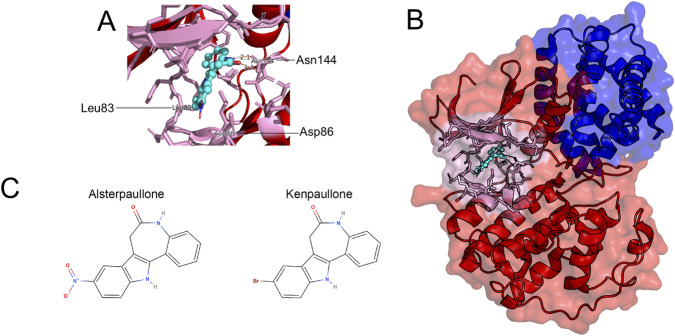
Structural basis of CDK5/p25 inhibition by indolobenzazepinones. **(A)** Close-up view of the ATP-binding pocket of the CDK5/p25 complex (chain A: red; chain D: blue) with Alsterpaullone (cyan) bound. Key interactions are mediated by Asp86 (pink sticks), forming hydrogen bonds (orange lines, distances in Å) with the inhibitor’s carbonyl and NH groups. The surrounding protein surface is shown semi-transparently in pink to highlight the binding cleft. **(B)** Global view of the CDK5/p25–Alsterpaullone complex. Chain A (CDK5) is rendered as red ribbons, chain D (p25) as blue ribbons, and the molecular surface is colored pink to delineate the active site region. Alsterpaullone (cyan) occupies the canonical ATP-binding site between the N- and C-terminal lobes, consistent with its mechanism as a competitive kinase inhibitor. **(C)** Chemical structures of two representative indolobenzazepinones-based CDK inhibitors: Alsterpaullone, Kenpaullone. 3D model created using SwissDock.

Alsterpaullone (Figures 3A–C), also termed 9-nitropaullone (NSC 705701, CAS 220202–03-4), is a prototypical paullone-class CDK inhibitor derived from kenpaullone optimization. Its tetracyclic 7,12-dihydroindolo [3,2-d][1]benzazepin-6(5H)-one core enables competitive ATP-site binding *via* canonical hinge hydrogen bonds, an interaction mode experimentally resolved in GSK-3β (PDB: 1Q3W) and structurally conserved in CDKs (e.g., with Leu83), while the 9-nitro group extends toward the phosphate-binding region (Gussio et al., 2004). This scaffold delivers potent low nanomolar inhibition of CDK1/cyclin B (IC_50_ = 35 nM), CDK2/cyclin A (IC_50_ = 15 nM), CDK2/cyclin E (IC_50_ = 200 nM), and CDK5/p25 (IC_50_ = 40 nM), alongside exceptional GSK-3α/β potency (IC_50_ = 4 nM) but weaker activity against CDK4/6 (>1 μM) and limited kinome selectivity (off-targets including CK1, CK2 at <1 μM), reflecting paullones’ dual CDK/GSK-3 pharmacology (PubChem, 2005b; Alsterpaullone, 2026; Alsterpaullone|CDK inhibitor|CAS 237430-03-4|Selleck, n.d.; Alsterpaullone (9-Nitropaullone)|CDK Inhibitor|MedChemExpress, n.d.). Functionally, it enforces G1/S and G2/M arrest, Rb hypophosphorylation, caspase activation, and apoptosis in cancer cells (GI_50_ = 0.5–5 μM in neuroblastoma, medulloblastoma, and breast lines), with preclinical efficacy in xenograft models at 5–20 mg/kg IP/oral doses, though overshadowed by GSK-3-mediated neurotoxicity and poor pharmacokinetics that halted clinical pursuit (Greenwald et al., 2004; Alsterpaullone|CDK inhibitor|CAS 237430-03-4|Selleck, n.d.). Alsterpaullone’s polypharmacology informed subsequent isoform-selective efforts like pyrazolopyrimidines, with IP claims in early paullone patents (e.g., WO1999026929 equivalents by Meijer et al.) encompassing nitro-substituted indolones for CDK/GSK-3 inhibition in proliferative and neurodegenerative contexts (Brugnara et al., 1999; Schultz et al., 1999; Leost et al., 2000; Knockaert et al., 2002; Alsterpaullone|CDK inhibitor|CAS 237430-03-4|Selleck, n.d.; Alsterpaullone (9-Nitropaullone)|CDK Inhibitor|MedChemExpress, n.d.).

Kenpaullone (Figure 3C), systematically 9-bromo-7,12-dihydroindolo [3,2-d]benzazepin-6(5H)-one (CAS 142273–20-9), represents the archetypal paullone-class CDK/GSK-3 dual inhibitor originating from natural product paullone modifications at CNRS Gif-sur-Yvette, characterized by a tetracyclic 7,12-dihydroindolo [3,2-d]benzazepin-6-one core bearing a 9-bromo substituent that enhances ATP-competitive binding through hinge hydrogen bonds (binding mode resolved in GSK-3β, PDB: 1UV5, and predicted to engage the conserved Leu83 hinge in CDKs) and contacts adjacent to the phosphate-binding region (Gussio et al., 2004; Kenpaullone, 2026; Kenpaullone|Glycogen Synthase Kinase 3|Tocris Bioscience, n.d.; PubChem, 2005c). This scaffold affords potent submicromolar inhibition of CDK1/cyclin B (IC_50_ = 400 nM), CDK2/cyclin A (IC_50_ = 680 nM), CDK2/cyclin E (IC_50_ = 700 nM), and CDK5/p25 (IC_50_ = 850 nM), complemented by exceptional GSK-3β potency (IC_50_ = 23–230 nM) but negligible activity against CDK4/6 (>10 μM) and moderate kinome selectivity (off-targets like CK1/CK2 at 1–5 μM), defining paullones’ signature polypharmacology (Kenpaullone, 2026; Kenpaullone|CDK inhibitor|CAS 142273-20-9|Selleck, n.d.). It potently inhibits CDK5/p25 and GSK-3β alongside CDK1/2, eliciting G1/S arrest, Rb hypophosphorylation, and β-catenin stabilization in cancer cells (GI_50_ = 1–10 μM in neuroblastoma, GBM, and stem-like lines). While CDK5 inhibition contributes to apoptosis and stem cell reprogramming (replacing Klf4), mechanism-driven neurotoxicity from combined GSK-3β/CDK5 disruption, alongside suboptimal ADME, ultimately precluded clinical development (Leost et al., 2000; Kitabayashi et al., 2019; Reinhardt et al., 2019).

### 2.4 Indirubin derivatives

**FIGURE 4:**
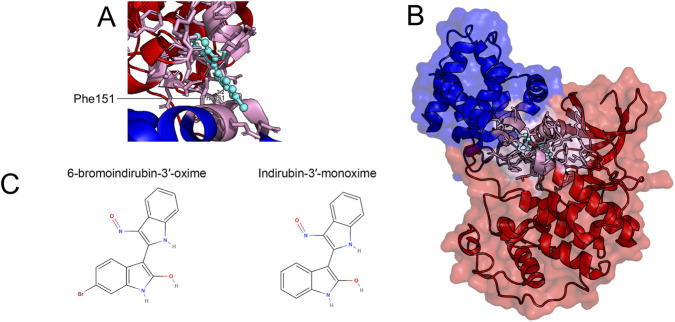
Structural basis of CDK5/p25 inhibition by indirubin derivatives. **(A)** Close-up view of the ATP-binding pocket of the CDK5/p25 complex (chain A: red; chain D: blue) with BIO (6-bromoindirubin-3′-oxime, cyan) bound. The inhibitor forms hydrogen bonds (orange lines, distances in Å) with key hinge residue Leu83 (pink sticks). The surrounding protein surface is rendered semi-transparently in pink to highlight the binding cleft. **(B)** Global view of the CDK5/p25–BIO complex. Chain A (CDK5) is depicted as red ribbons, chain D (p25) as blue ribbons, and the molecular surface is shown in pink to delineate the active site region. BIO (cyan) occupies the canonical ATP-binding site between the N- and C-terminal lobes, consistent with its mechanism as a competitive kinase inhibitor targeting the hinge region. **(C)** Chemical structures of two representative indirubin-based CDK inhibitors: BIO (6-bromoindirubin-3′-oxime), Indirubin-3′-monoxime. 3D model created using SwissDock.

Indirubin-3′-monoxime (Figure 4C), a key derivative of the natural bis-indole alkaloid indirubin, is a potent ATP-competitive inhibitor targeting CDKs, notably CDK1, CDK2, and CDK5, as well as glycogen synthase kinase-3 (GSK-3). Structurally, it consists of an indirubin core modified by the introduction of a monoxime group at the 3′ position, enhancing solubility and kinase inhibitory potency compared to indirubin itself (Jautelat et al., 2003; PubChem, 2006a). Crystallographic studies of indirubin derivatives bound to CDK2 (PDB: 2BHE), CDK5/p25 (PDB: 1UNH), and GSK-3β (PDB: 1Q41) reveal a conserved binding mode in the ATP pocket, anchored by hydrogen bonds to the hinge region (e.g., Leu83 in CDK2) and stabilized by hydrophobic contacts (Yan et al., 2015; Indirubin-3’-monoxime|CDK inhibitor|CAS 160807-49-8|Selleck, n.d.). Functionally, indirubin-3′-monoxime induces cell cycle arrest, inhibits phosphorylation of substrates such as Rb and tau proteins, and promotes apoptosis in cancer and neurodegenerative disease models. Despite promising preclinical efficacy, including anti-proliferative and neuroprotective effects, its clinical use has been limited by poor pharmacokinetic properties and selectivity concerns due to broad kinase inhibition (Yan et al., 2015; Reinhardt et al., 2019).

BIO (6-bromoindirubin-3′-oxime) (Figures 4A–C) is a brominated indirubin derivative characterized by a bromine atom substitution at the 6-position and a 3′-oxime modification on the indirubin scaffold. This modification improves potency and kinase binding affinity over the parent indirubin compounds (Meijer et al., 2011; Sklirou et al., 2017). BIO acts as a potent ATP-competitive inhibitor predominantly targeting cyclin-dependent kinases CDK1, CDK2, and CDK5, as well as glycogen synthase kinase-3 (GSK-3), with nanomolar IC_50_ values typically in the range of 10–50 nM across these kinases. Structurally, BIO engages the kinase ATP-binding pocket through critical hydrogen bonding with hinge residues (e.g., Leu83 in CDK2), and its bromine substituent facilitates hydrophobic contacts within the kinase active site, enhancing binding affinity (Sklirou et al., 2017; Guo et al., 2019). Functionally, BIO induces robust inhibition of CDK1/2/5- and GSK-3β-mediated phosphorylation events, leading to cell cycle arrest and apoptosis in multiple cancer cell lines, and modulation of Wnt/β-catenin and neuronal signaling pathways, with noted effects in stem cell biology and neurodegeneration models (Martelli et al., 2022; Shareena et al., 2023). However, like other indirubin derivatives, BIO’s clinical translation has been limited by suboptimal selectivity and pharmacokinetic challenges, driving ongoing efforts to refine its chemical properties for therapeutic use (Kitabayashi et al., 2019; Reinhardt et al., 2019; Kornsuthisopon et al., 2022).

### 2.5 Other scaffolds

**FIGURE 5:**
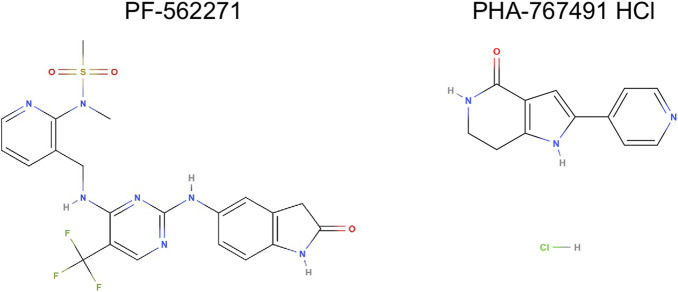
Structure of non-canonical scaffold CDK inhibitors: PF-562271, PHA-767491 HCl (right).

PHA-767491 HCl (Figure 5, right), also known as CAY10572 or NMS-1116354 (CAS 942425–68-5), exemplifies a dual Cdc7/CDK9 inhibitor developed by Nerviano Medical Sciences, featuring a 2-(pyridin-4-yl)-1,5,6,7-tetrahydropyrrolo [3,2-c]pyridin-4-one core that binds ATP-competitively with hinge hydrogen bonds (binding mode resolved in CDC7, PDB: 4F9B, and predicted to engage the conserved Leu83-equivalent hinge in CDKs) and hydrophobic contacts mediated by the fused pyrrolopyridinone scaffold (Vanotti et al., 2008; PHA-767491 hydrochloride (CAY-10572 hydrochloride)|CDK Inhibitor|MedChemExpress, n.d.). This scaffold yields potent low nanomolar inhibition of Cdc7 (IC_50_ = 10 nM) and CDK9 (IC_50_ = 34 nM), with modest selectivity (∼20-fold over CDK1/2, ∼50-fold over CDK5/MK2, >100-fold over PLK1/CHK2/GSK3β in cell-free assays), though cross-reactivity contributes to broad antiproliferative effects. Functionally, it blocks DNA replication initiation (*via* Cdc7-Dbf4), suppresses Mcl-1 expression and RNAP II CTD phosphorylation (*via* CDK9), induces S-phase arrest, caspase activation, and apoptosis (GI_50_ = 0.1–1 μM in HCC, leukemia, and solid tumor lines), with synergy alongside 5-FU in xenografts by counteracting Chk1 phosphorylation (Natoni et al., 2009; Pauzaite et al., 2022). Despite promising preclinical antitumor activity and oral bioavailability, clinical progression stalled post-phase I due to toxicity and overlapping profiles with isoform-selectives; its hydrochloride salt aids solubility, and IP resides in Nerviano patents (e.g., WO2009138635 equivalents) claiming pyrrolopyridinones for replication/transcriptional CDK modulation (Natoni et al., 2009; Alzani et al., 2010).

PF-562271 (also known as VS-6062) (Figure 5, left) and its salts, including PF-00562271 Besylate and PF-562271 HCl, are potent, selective, ATP-competitive, and reversible inhibitors of focal adhesion kinase (FAK) and the related kinase PYK2. Chemically, PF-562271 features a trifluoromethyl substituted bis-amino pyrimidine core, stabilizing a distinctive DFG-in helical conformation in the kinase domain, which contributes to its selectivity profile (Kath et al., 2012). PF-562271 exhibits highly potent inhibition of FAK with an IC_50_ of approximately 1.5 nM and PYK2 at around 13 nM, effectively blocking phosphorylation of FAK at Tyr397 and downstream signaling cascades that regulate cell adhesion, migration, proliferation, and survival (Wiemer et al., 2013; PubChem, 2006b; PF-562271 (VS-6062)|FAK/Pyk2 Inhibitor|MedChemExpress, n.d.). PF-562271 has emerged in biochemical and cellular studies as a moderate inhibitor of CDK5, exhibiting an IC_50_ of approximately 120 nM in recombinant enzyme assays. This off-target activity positions CDK5 among a cluster of secondary targets including CDK1, CDK2, CDK3, and CDK7 (IC_50_ = 30–120 nM), as consistently documented in comprehensive kinome profiling by commercial vendor. In cellular contexts, PF-562271 demonstrates functional CDK5 engagement at concentrations of 1–5 μM, where it attenuates CDK5-dependent phosphorylation of FAK at Serine 732, a key marker of kinase activity that regulates focal adhesion dynamics and cytoskeletal remodeling (Verastem, Inc, 2013; Ortiz-Rivera et al., 2023). This dual inhibition manifests in GBM (GL261) and pancreatic cancer (BxPC-3) models, where PF-562271 suppresses cell migration and invasion by 60%–80% while achieving 86% tumor growth inhibition in BxPC-3 xenografts upon oral dosing (50 mg/kg twice daily), effects attributable in part to synergistic FAK/CDK5 blockade beyond canonical FAK signaling alone (Stokes et al., 2011; Rolon-Reyes et al., 2015; Ortiz-Rivera et al., 2023). Neurodegenerative repurposing potential has also been explored in TauP301S neuronal models, where 10 μM exposure reduces pathological tau phosphorylation at Serine 396/404 by 45%, suggesting applicability in tauopathies despite suboptimal blood-brain barrier penetration (log_10_P ≈3.5). While PF-562271 is a selective FAK/Pyk2 inhibitor, the concentration used (10 μM) exceeds its reported CDK5/p25 IC_50_ in broad kinase panels, raising the possibility that off-target CDK5 inhibition may contribute to reduced tau phosphorylation. These properties make PF-562271 and its salts valuable chemical probes for dissecting focal adhesion and related signaling pathways, though mechanistic deconvolution requires orthogonal validation (Wiemer et al., 2013; Hu et al., 2024; PubChem, 2006b; PF-562271 (VS-6062)|FAK/Pyk2 Inhibitor|MedChemExpress, n.d.).

### 2.6 Peptides

CDK5 inhibitory peptide (CDK5i), encompassing SEQ ID NO: 2 (ARAFGIPVRCYS) and its cell-penetrating derivative SEQ ID NO: 3 (ARAFGIPVRCYSYGRKKRRQRRR), represents a designed peptide therapeutic targeting pathological CDK5 hyperactivity. Chemically, the core inhibitor comprises a 12-amino acid sequence derived from the CDK5 p25-binding interface, optionally linked to a TAT domain to facilitate BBB translocation and cellular uptake. CDK5i exhibits a distinctive mechanism of action by selectively disrupting the CDK5-p25/p35 protein-protein interaction without inhibiting basal CDK5 activity mediated by p35, thereby preserving physiological kinase function essential for neurodevelopment. This selectivity profile is further underscored by a lack of binding affinity for homologous kinases CDK1 and CDK2, as confirmed by pull-down assays in brain lysates. In human familial AD iPSC-derived neural progenitor cells, CDK5i treatment at 1 μM significantly reduces levels of HDAC2 and γH2AX, markers associated with transcriptional repression and DNA damage respectively. *In vivo* efficacy was demonstrated in P301S mouse models of AD and frontotemporal dementia, where intraperitoneal administration (40 mg/kg) resulted in detectable brain concentrations *via* targeted mass spectrometry and significant reduction of CDK5 kinase activity in brain lysates without affecting wild-type basal activity. These properties position CDK5i as a highly specific chemical probe and candidate therapeutic for cognitive function disorders, including AD, Huntington’s disease, and frontotemporal dementia. This offers a strategy to mitigate neurodegeneration while avoiding the toxicity associated with pan-CDK inhibition (Tsai and Seo, 2025).

## 3 Clinical and preclinical challenges

Obstacles in CDK5 inhibition include off-target effects on other CDKs. Many current CDK inhibitors that affect CDK5 also inhibit multiple other CDK family members such as CDK1, CDK2, CDK7, and CDK9, leading to multi-kinase inhibition which can cause adverse effects including myelosuppression and organ toxicities. Since CDK5 plays critical physiological roles in neuronal functions, its broad inhibition can disrupt normal cellular processes (Khair et al., 2019; Allnutt et al., 2020; Umfress et al., 2022; Tang et al., 2023; Alrouji et al., 2024).

Most preclinical CDK5 inhibitors display limited tissue penetrance, especially crossing the BBB to effective levels without systemic toxicity. In clinical trials with pan-CDK inhibitors like roscovitine and dinaciclib, toxicities associated with off-target CDK inhibition have limited their efficacy. Development of inhibitors that precisely target CDK5 while minimizing activity against other CDKs remains a challenge. Efforts continue to engineer highly selective, brain-penetrant CDK5 inhibitors to reduce these side effects and improve clinical efficacy (Khair et al., 2019; Umfress et al., 2022; Tang et al., 2023; Alrouji et al., 2024).

Another challenge is the lack of validated biomarkers for CDK5 activity. Unlike other CDKs where phosphorylation status of known substrates or kinase activity can be directly measured, CDK5’s diverse roles especially in neuronal cells, and its regulation by non-cyclin activators like p35 and p25, complicate direct assessment of inhibition (Khair et al., 2019; Do and Lee, 2021). Indirect markers such as phosphorylation levels of CDK5 substrates are used in preclinical studies to infer target engagement. For example, CDK5 can phosphorylate pAkt at Serine 473 and retinoblastoma protein phosphorylation at Serine 780 (Sharma and Sicinski, 2020; Zhou et al., 2021). Another known substrate for CDK5 is FAK, and its phosphorylation at Serine 732 can be used as a reliable marker of CDK5 activity (Pozo and Bibb, 2016; Sharma and Sicinski, 2020). Additionally, CDK5 can autophosphorylate at Serine 159, which can be used as activity indicator (Sharma et al., 1999; Zhang et al., 2014). However, these are context-dependent and can be influenced by parallel pathways, thus limiting specificity. Moreover, CDK5’s activity changes dynamically with its activators and cellular context, making a universal and robust biomarker unlikely (Shupp et al., 2017; Khair et al., 2019; Allnutt et al., 2020; Do and Lee, 2021; Pao et al., 2023).

Newer approaches include development of peptide-based CDK5 inhibitors that are intended to more selectively modulate pathological CDK5 signaling and may facilitate biomarker discovery (Tsai and Seo, 2025). Circulating CDK5 levels or activity-related peptides are being investigated as potential biomarkers, particularly in neurodegenerative diseases. Overall, the absence of well-validated biomarkers hampers clinical translation, complicating dose optimization and efficacy monitoring of CDK5 inhibitors. Continued research into selective substrates, phosphorylation signatures, and complex-specific inhibitors is critical to overcome this challenge (Khair et al., 2019; Batra et al., 2023; Pao et al., 2023; Kumari et al., 2025). Complementary to occupancy-driven inhibition, proteolysis-targeting chimeras (PROTACs) have emerged as a promising modality for CDK5. By hijacking the ubiquitin–proteasome system, PROTACs enable event-driven degradation of CDK5, potentially bypassing active-site competition, targeting non-catalytic scaffolding functions, and overcoming compensatory resistance mechanisms. Preclinical studies demonstrate that CDK5-directed PROTACs achieve sustained pathway suppression in neurodegenerative and oncological models, with early evidence of improved isoform selectivity over pan-CDK inhibitors (Teng et al., 2020; Riching et al., 2021; Neerasa et al., 2025). Nevertheless, clinical translation requires optimization of blood–brain barrier permeability, tissue-selective E3 ligase recruitment, and degradation-specific pharmacodynamic biomarkers. Integrating PROTAC development with activity-based profiling and phospho-signature mapping may accelerate target validation and dose optimization in future trials (Tashima, 2023; Yokoo et al., 2023).

CDK5 inhibition testing in animal models does not recapitulate human disease, primarily due to differences in CDK5 regulation, brain physiology, and pathology. Whereas MPTP-induced Parkinson’s or CK-p25 transgenic AD models effectively demonstrate CDK5/p25 hyperactivation and neuroprotection from inhibitors, these models inadequately capture human tauopathy progression, amyloid-beta accumulation kinetics, and chronic neuroinflammation (Menn et al., 2010; Zhang et al., 2013; Allnutt et al., 2020; Ao et al., 2022; Umfress et al., 2022; Paul et al., 2025). Additionally, animal models often neglect CDK5’s physiological roles in adult neurogenesis and synaptic plasticity, masking on-target toxicity (Menn et al., 2010; Umfress et al., 2022). Major explanations for such discrepancy include rodents’ accelerated pathology [e.g., neurofibrillary tangle formation in weeks vs. decades in humans (AlShimemeri et al., 2021)], underdeveloped BBB penetration mimicking human pharmacokinetics, and compensatory mechanisms absent in humans, leading to overestimation of efficacy [e.g., (S)-roscovitine’s stroke neuroprotection in rats not replicating clinically (Menn et al., 2010; Rousselet et al., 2018)].

The delivery of CDK5 inhibitors to the CNS is limited by BBB, which restricts >98% of small molecules owing to tight endothelial junctions and efflux transporters (e.g., P-gp). The BBB has stringent physicochemical requirements like low molecular weight (<400 Da), log_10_P = 1–3, and minimal hydrogen bond donors for any compounds to penetrate through it. Many CDK5 inhibitors are characterized by poor aqueous solubility, high polarity, or substrate status for ABC transporters, which results in low brain/plasma ratios (QPlogBB < −1) and insufficient free brain exposure for sustained p35/p25 inhibition (Menn et al., 2010; Umfress et al., 2022; Yang, 2025). Preclinical studies highlight injectable formulations (e.g., pH-adjusted cyclodextrin complexes for (S)-roscovitine) improving solubility but often failing chronic oral dosing due to first-pass metabolism and variable BBB penetration in rodents vs. primates/humans. Off-target CDK inhibition exacerbates toxicity at higher systemic doses needed for CNS efficacy, whereas neuronal localization of CDK5 demands targeted delivery to avoid peripheral effects (Menn et al., 2010; Zeb et al., 2019; Allnutt et al., 2020; Umfress et al., 2022). Novel solutions include lipid nanoparticles, BBB-shuttling peptides [e.g., angiopep-2 (Dong et al., 2019; Anami et al., 2022)], or prodrug strategies enhancing lipophilicity and transporter evasion, as seen in computational designs crossing BBB to abrogate tau pathology. Despite progress, no CDK5 inhibitor has cleared clinical CNS hurdles, underscoring needs for human iPSC-BBB *in vitro* models and PET-tracers for PK/PD optimization (Zeb et al., 2019; Allnutt et al., 2020; Dhariwal et al., 2025; V et al., 2025; Yang, 2025).

Future research in the field should consider using human iPSC-derived neuronal models for target validation, non-human primate studies for PK/PD correlation, and pharmacodynamic biomarkers (e.g., CSF p-Tau or p35/p25 ratios) (Robin et al., 2017; Allnutt et al., 2020; Ao et al., 2022).

## 4 Conclusion

The patent landscape for CDK5 inhibitors (Table 1) reflects growing interest in targeting this kinase in neurodegenerative disorders and cancer. A diverse set of chemotypes has been disclosed, including purine analogs (Roscovitine, Purvalanol A & B), indolobenzazepinones (Alsterpaullone, Kenpaullone), indirubin derivatives (Indirubin-3′-monoxime, BIO), pyrazole derivatives (Dinaciclib, PHA-793887, Milciclib, AT7519, PHA-767491 HCl), and other scaffolds (PF-562271). Recent innovations include peptide-based agents, such as the CDK5 inhibitory peptides disclosed in the recent 2025 US12384818B2, which aim to modulate pathological CDK5 signaling in cognitive impairment. Despite these advances, achieving true selectivity over other CDKs, especially CDK1, CDK2, and GSK3β, remains a major problem due to high ATP-site homology, and many patented small-molecule inhibitors still display pronounced polypharmacology that, while potentially advantageous in oncology, complicates mechanistic interpretation and elevates off-target risk. Current efforts focus on improving brain penetrance for neurological applications, enhancing kinase selectivity, and exploring non-ATP-competitive mechanisms. Continued innovation in structure-guided design and biomarker-driven development will be critical to advancing viable candidates into the clinic.

**TABLE 1:** Overview of CDK5 inhibitors in literature and clinical development.

Inhibitor	Chemotype/Class	Primary target(s)	Selectivity notes	Development/Use context
Roscovitine	Purine analog	CDK5, CDK2, CDK7, CDK9	Low selectivity; pan-CDK inhibitor	Phase II trials (cancer, neurodegeneration) (Bettayeb et al., 2010; Meijer et al., 2022)
Purvalanol A, B	Purine analog	CDK5, CDK2	More potent than roscovitine but still non-selective	Preclinical tool compound (Ringer et al., 2010; P-2481-10MG - Purvalanol A, 10 MG, n.d.; Purvalanol B (NG 95)|CDK Inhibitor|MedChemExpress, n.d.)
Dinaciclib	Pyrazole derivative	CDK5, CDK2, CDK1, CDK9	Potent multi-CDK inhibitor (IC_50_ = 1 nM for CDK5)	Phase I trials (leukemia, solid tumors) (Johnson et al., 2012; Flynn et al., 2015; Oner et al., 2025)
PHA-793887	Pyrazole derivative	CDK5, CDK2, CDK1	ATP-competitive; nanomolar potency	Preclinical oncology studies (Alzani et al., 2010; Verastem, Inc., 2013; PHA-793887|CDK inhibitor|CAS 718630-59-2|Selleck, n.d.)
AT7519	Pyrazole derivative	CDK5, CDK2, CDK1, CDK4, CDK9	Multi-CDK inhibitor; limited oral bioavailability	Phase I/II trials (hematologic malignancies) (Santo et al., 2010; Chen et al., 2014; Zhao et al., 2023)
Milciclib	Pyrazole derivative	CDK5, CDK2, CDK4, CDK7	Orally bioavailable; modest selectivity	Phase II trials (hepatocellular carcinoma) (Gussio et al., 2004; Villa et al., 2020)
Alsterpaullone	Indolobenzazepinone	CDK5, GSK3β	Moderate CDK5 potency; also inhibits GSK3β	Preclinical research (Lahusen et al., 2003; Alsterpaullone|CDK inhibitor|CAS 237430-03-4|Selleck, n.d.)
Kenpaullone	Indolobenzazepinone	CDK5, CDK1	Similar scaffold to alsterpaullone	Preclinical research (Kitabayashi et al., 2019; Yeo et al., 2021; Smail et al., 2026)
Indirubin-3′-monoxime	Indirubin derivative	CDK5, GSK3β	Natural product derivative; moderate selectivity	Widely used in neuroscience research (Kim et al., 2021; Fang et al., 2025; PubChem, 2017)
BIO	Indirubin derivative	CDK5, GSK3β	Potent GSK3β inhibition; cell-permeable	Preclinical tool compound (Guo et al., 2019; GSK 3 Inhibitor IX (6-Bromoindirubin-3’-oxime)|ATP-Competitive Inhibitor|MedChemExpress, n.d.)
PHA-767491 HCl	Other scaffolds	CDK5, CDK9, FAK	Originally developed as FAK inhibitor	Preclinical cancer models (Natoni et al., 2009; Pauzaite et al., 2022)
PF-562271	Other scaffolds	FAK > CDK5 (weak)	Primarily a FAK inhibitor; minor CDK5 activity	Repurposed/optimized in CDK5 studies (Wiemer et al., 2013)
CDK5 inhibitory peptides	Peptide (ARA-FGX_1_-PVRCX_2_S-derived)	CDK5	Sequence-defined CDK5 inhibition; proposed to modulate aberrant CDK5 signaling rather than broad pan-CDK blockade	Preclinical; patented for enhancement of cognitive function and treatment of cognitive disorders (Tsai and Seo, 2025)
